# The Relationship Between Skeletal-Related Events and Bone Scan Index for the Treatment of Bone Metastasis With Breast Cancer Patients

**DOI:** 10.1097/MD.0000000000000269

**Published:** 2014-12-02

**Authors:** Toshiaki Iwase, Naohito Yamamoto, Hironori Ichihara, Takashi Togawa, Takeshi Nagashima, Masaru Miyazaki

**Affiliations:** From the Division of Breast Surgery (TI, NY); Division of Nuclear Medicine (HI, TT), Chiba Cancer Center, Japan; and Department of General Surgery (TI, TN, MM), Chiba Graduate School of Medicine, Japan.

## Abstract

The aim of the present study was to investigate the relationships between the automated bone scan index (aBSI) and skeletal-related events (SRE) in breast cancer patients with bone metastasis. A computer-aided software (BONENAVI™) that was developed using an Artificial Neural Network (Artificial Neural Network) was used for the present analysis.

Forty-five patients diagnosed with bone metastasis due to breast cancer from April 2005 through March 2013 were retrospectively analyzed. Before and after the time of initial treatment, aBSI, Artificial Neural Network score, and hotspot number were calculated, and the relationships between these scores and SRE were analyzed.

Twenty cases showed decreased (improved) aBSI values after initial treatment (Group A), and 25 cases showed unchanged/increased (worsened) aBSI values (Group B). Chi-square analysis revealed a significant difference in incident numbers of SRE between the two groups—one case in Group A and 12 in Group B (*P* < 0.001). Event-free survival was significantly shorter in Group B (hazard ratio: 8.31, 95% CI: 1.33–12.14, log-rank test; *P* < 0.05). The groups were also divided by the results of 2 radiologists’ visual scan interpretations, and no significant differences were shown in the number of SRE (*P* = 0.82, *P* = 0.10). When correlation analyses were performed between aBSI and bone metabolic or tumor markers, alkaline phosphatase was significantly correlated with aBSI at the time of initial treatment (*R* = 0.69, *P* < 0.05).

In conclusion, aBSI is proposed as a useful and objective imaging biomarker in the detection of breast-cancer patients with bone metastasis at high risk of SRE.

## INTRODUCTION

Recently, newly developed molecular agents targeted against receptor activator of nuclear factor κ-B ligand have shown remarkable outcomes in preventing skeletal-related events (SRE) in breast cancer patients with bone metastasis.^1^ On the other hand, few biomarkers evaluating the extent of bone metastasis have been developed.

Bone scan index (BSI) is a new imaging biomarker that was originally reported by Erdi et al at the Memorial Sloan Kettering Cancer Center in 1997. The BSI evaluates the range of bone metastasis—“hot spots” are expressed as a percentage of total bone amount.^2^ BSI was originally calculated manually, but recently the automated BSI (aBSI) has been developed by imitating an Artificial Neural Network (Artificial Neural Network), such as in the human brain. Therefore, BSI has become a more convenient tool.^3,4^ While bone scintigraphy has been widely used to evaluate bone metastasis for a long time and allows visual interpretation of the metastatic site, quantitative evaluation of bone metastasis on a bone scan requires certain skills. In contrast, aBSI is an objective quantitative measure.

In the American Society of Clinical Oncology Clinical Practice Guideline, routine bone scans in non-symptomatic breast cancer patients are not recommended on the basis of negative reported opinions.^5^ On the other hand, the aim of bone metastasis treatment has recently shifted to the reduction of SRE, and reconsideration of the usefulness of the bone scan has been suggested, particularly for patients with a high risk for SRE.^6^ The role of the bone scan is set to change in the near future to a means of obtaining an imaging biomarker to help reduce SRE.

The usefulness of the aBSI as an imaging biomarker has been previously investigated in prostate cancer, but only sporadically in bone metastasis treatment in breast cancer.^7,8^ The relationship between aBSI and SRE in breast cancer warrants renewed analysis because the manifestation of bone metastasis in breast cancer differs from that in prostate cancer.

The aim of this study was to investigate the usefulness of the aBSI as an imaging biomarker in bone metastasis treatment in breast cancer.

## MATERIALS AND METHODS

### Patient Selection

Among 97 patients diagnosed with metastatic breast cancer by undergoing a core biopsy or surgery from April 2005 through March 2013, 45 matched according to the following criteria were included in the study: bone metastasis detected at the initial outpatient examination on bone scan or computed tomography (27 cases), or during the follow-up period after surgery (18 cases), and bone scan performed with methylene-diphosphonic acid technetium (MDP) before and after treatment. The MDP bone scan images were required because BONENAVI™ requires 99mTc images for matching with its own database. If applicable, the detection of bone metastasis manifesting within the treatment period was recorded according to the order of its appearance relative to that of other metastatic sites (eg, liver and lung). Ethical approval was obtained for this study from the Ethical Board of the institutional review board.

### Bone Scintigraphy Procedure

**FIGURE 1:**
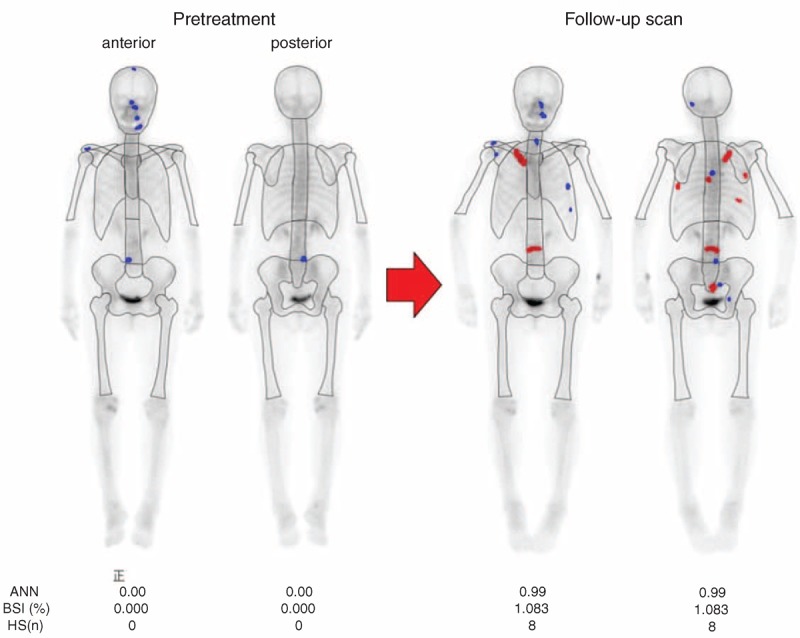
aBSI: aBSI reflects the burden of the skeleton. The tumor burden is expressed as a percentage of the total skeletal mass. ANN: Each individual hotspot was classified as metastasis or not. The possibility of metastasis was expressed by the continuous ANN number, ranging from 0 to 1.

The bone scan devices used for the present study were dual-head nuclear gamma camera systems (GCA 7200A/UI and eCAM; TOSHIBA, Co. Ltd., Tokyo, Japan). 740 mBq (20 mCi) Technetium-99m methylene diphosphonate (Tc99m-MDP) was given intravenously. The low energy high-resolution collimator was selected, and scanning was performed 2 hours after the administration. During scanning, the patients were supine position and dual-head anterior and posterior whole body images were obtained at 15 cm/min. Collected data were analyzed by a single nuclear physician (HI) using the computed-aided diagnosis software BONENAVI™ (FUJIFILM RI Pharma, Co. Ltd., Tokyo, Japan; EXINIbone, EXINI Diagnostics, Lund, Sweden). In addition to aBSI, the ANN and hot spot numbers were calculated. ANN predicts the possibility of bone metastasis in each individual hot spot by showing continuous numbers ranging from 0 to 1 by imitating a human neural network based on the Japanese database (Figure 1).^4^

### Follow-Up After Surgery

The patients were categorized according to pathological findings into three risk groups as advocated by the St. Gallen International Breast Cancer Congress in 2007.^9^ The follow-up period was set to 6 years; bone scans were performed in the intermediate group (second year and fifth year) and high-risk group (once a year until the fifth year) but not in the low-risk group. Serum biomarkers were measured twice a year until the fifth year regardless of the risk.

### Definition of SRE

The SRE were defined as follows: palsy, pathological fracture, radiation, and surgery. An oncologic orthopedic surgeon judged whether palsy was due to bone metastasis or other reasons, as well as diagnosed pathological fracture. A radiotherapist judged the necessity of radiotherapy. The dosage of radiotherapy was set to 30 gray, divided into 10 treatments, for pain control or palsy. Some patients received radiotherapy for mitigating urgent palsy arising from tumor compression of the spinal cord. Those cases were classified in the palsy group, and the radiation group included the patients that underwent radiotherapy for other reasons.

### Statistical Methods

Patients were divided into two groups according to the initial aBSI change with the bone metastatic treatment: Group A included patients with decreased aBSI values, and Group B included patients with unchanged/increased (worsened) aBSI values. The study patients were also divided into two groups (Group A: improved, Group B: unchanged/worsened) according to visual scan interpretations of two radiologists (HI and TT), and the aBSI results were compared with the radiologists’ results. To avoid information bias, the radiologists were blinded to information about SRE and aBSI results, and were required to read the images independently. The reproducibility between the readers was evaluated by Cohen Kappa statistic.

Incident numbers of SRE in each group were analyzed by chi-square test. The associations of a bone metabolic marker (alkaline phosphatase [ALP]), a cell injury marker (lactate dehydrogenase [LDH]), and tumor markers (carcino-embryo antigen [CEA] and carbohydrate antigen 15-3 [CA15-3]) with aBSI were also analyzed. Event-free survival (EFS) was defined as the period from initial diagnosis of bone metastasis to the incidence of SRE. When initial metastasis was diagnosed at distant organs such as the lung or liver, and bone metastasis was found as the second or third site during the treatment period, EFS was defined as the period from the time of bone metastasis to the onset of SRE. Overall survival (OS) was defined as the period from initial diagnosis of bone metastasis to death. Survival curves were compared by drawing the Kaplan–Meier curves, and log-rank tests were performed. All analyses were performed two-sided, and *P* < 0.05 was considered significant. GraphPad Prism5™ (GraphPad Software, Inc., La Jolla, CA, USA) was used as statistical software.

## RESULTS

### Patient Demographics

Thirty-one patients were diagnosed with bone metastasis as an initial recurrence after surgery. On the other hand, metastasis occurred first at other sites and then in bone in 4 patients: bone was the second site in 3 patients and the third in 1. In subtypes, 42 patients were estrogen positive and 12 were positive for human epidermal growth factor receptor-2 (HER2); only 1 patient was triple negative (TN), and bone was diagnosed as the third metastatic site. When SRE were stratified by subtype, there were 8 ER (+), HER2 (−) type cases, 3 ER (+), HER2 (+) type cases, and 1 case each in the HER2 (+) type and TN type, suggesting increased frequency in the ER (+), HER2 (−) type. Thirteen SRE were encountered, 5 palsy, 7 radiation, 1 surgery, 0 fractures. Five patients in the palsy group received radiation therapy for the purpose of prevention or palliative treatment, and those cases were counted as palsy, not radiation. Seven patients received radiation therapy for the purpose of pain control because of bone metastasis. Median aBSI before initiating treatment was 0.28 (range, 0.0–9.0). Bone modifying agents (BMA) were administered in 29 patients, zoledronic acid in 28 and denosumab in 1. Average follow-up period from initial bone scan to the first follow-up was 401 days, 505 days in group A and 324 days in group B (Table 1).

**TABLE 1:**
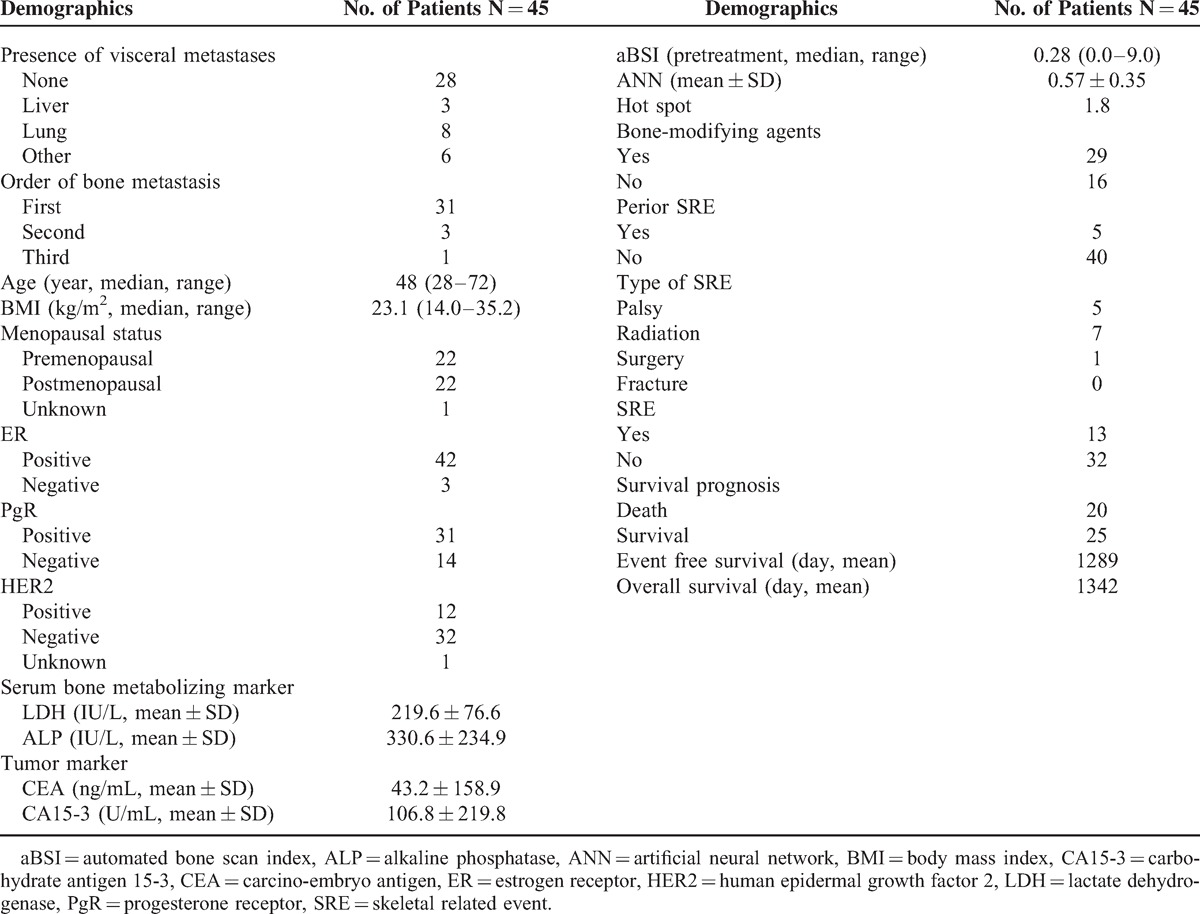
Patient Demographics

### SRE and aBSI Change

The number of cases in Group A was 20 and in Group B was 25. Median rate of change in each group was −54.2% (range, 0% to −95.8%) in Group A and 195.9% (100–6717.6%) in Group B. Univariate analysis showed no significant differences in patient demographics between Groups A and B. Thirteen patients experienced SRE during the follow-up period, but only 1 of these patients was in Group A. Univariate analysis showed significant differences in the incidence of SRE between the two groups (Table 2, chi-square test, *P* < 0.05). The patient in Group A who experienced SRE received emergency radiation therapy because of emerging palsy due to spinal cord compression by spinal bone metastasis.

**TABLE 2:**
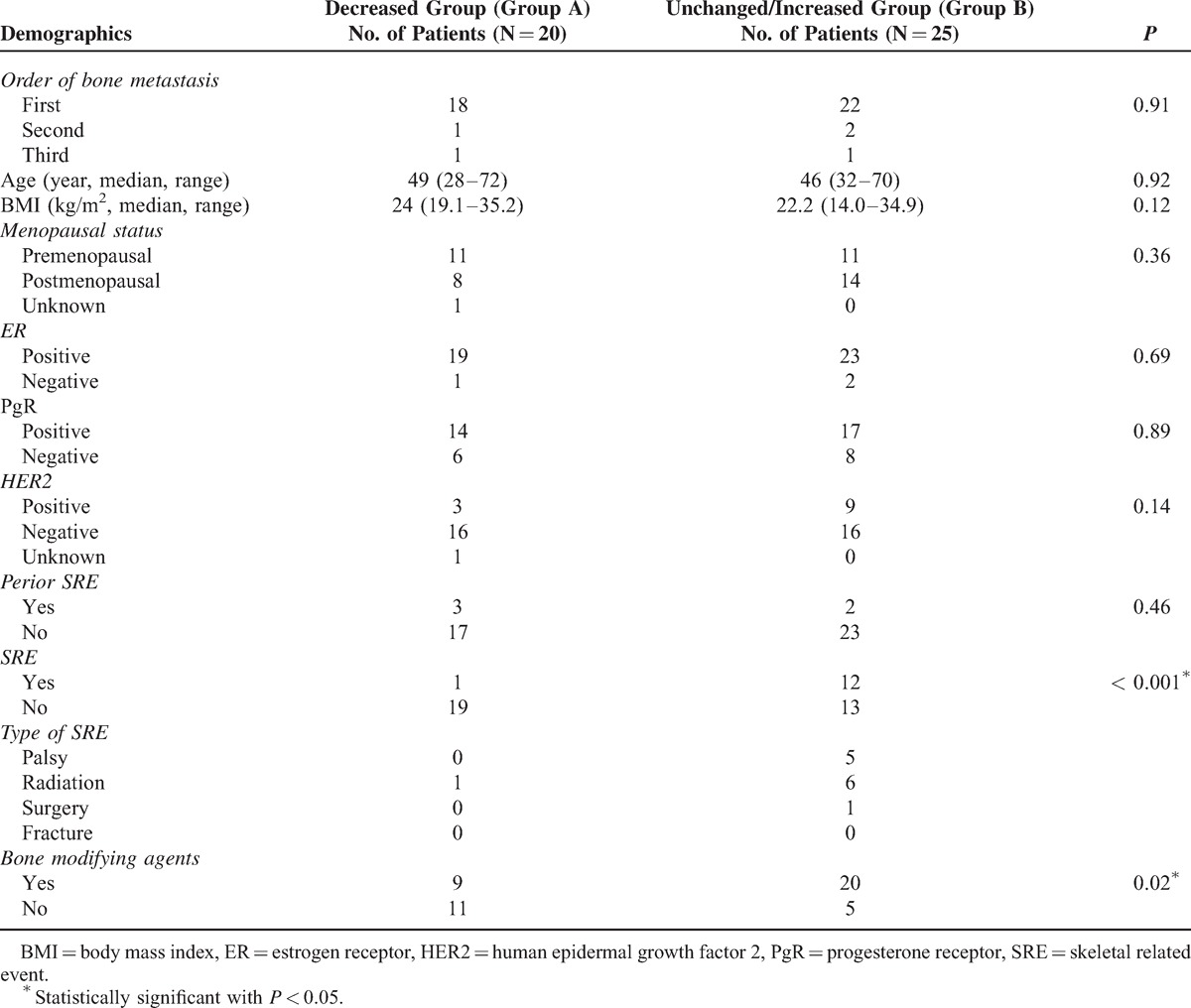
Result of Univariate Analysis between Decreased and Unchanged/Increased aBSI Group

### Visual Scan Interpretation

When the study patients were divided by the visual scan interpretations of two radiologists, Group A (improved)/Group B (unchanged/worsened) patient numbers were 11/34 (Radiologist A) and 12/33 (Radiologist B). High reproducibility between the readers was obtained as shown by Cohen Kappa statistic (κ = 0.731). Further, reproducibility between the aBSI and radiologist judgment was 0.645 for Radiologist A and 0.673 for Radiologist B; both these values were considered significantly coefficient.

### aBSI and Serum Biomarkers

**FIGURE 2:**
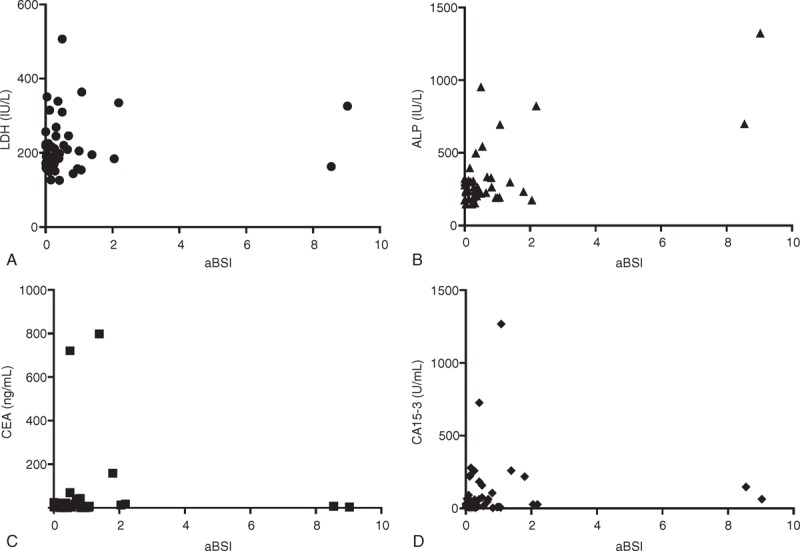
(A–D) Correlation analyses between a bone metabolic marker, cell injury marker, and tumor marker and aBSI at the time treatment started. Alkaline phosphatase appeared to have considerable correlation with the aBSI.

Correlation analyses between a bone metabolic marker, cell injury marker, and tumor marker and aBSI at the time treatment started yielded the following results: LDH, *R* = 0.12, *P* = 0.45; ALP, *R* = 0.69, *P* < 0.05; CEA, *R* = 0.01, *P* = 0.93; and CA15-3, *R* = 0.04, *P* = 0.79. From those results, ALP appeared to have considerable correlation with the aBSI (Figure 2A–D).

### EFS and Overall Survival

**FIGURE 3:**
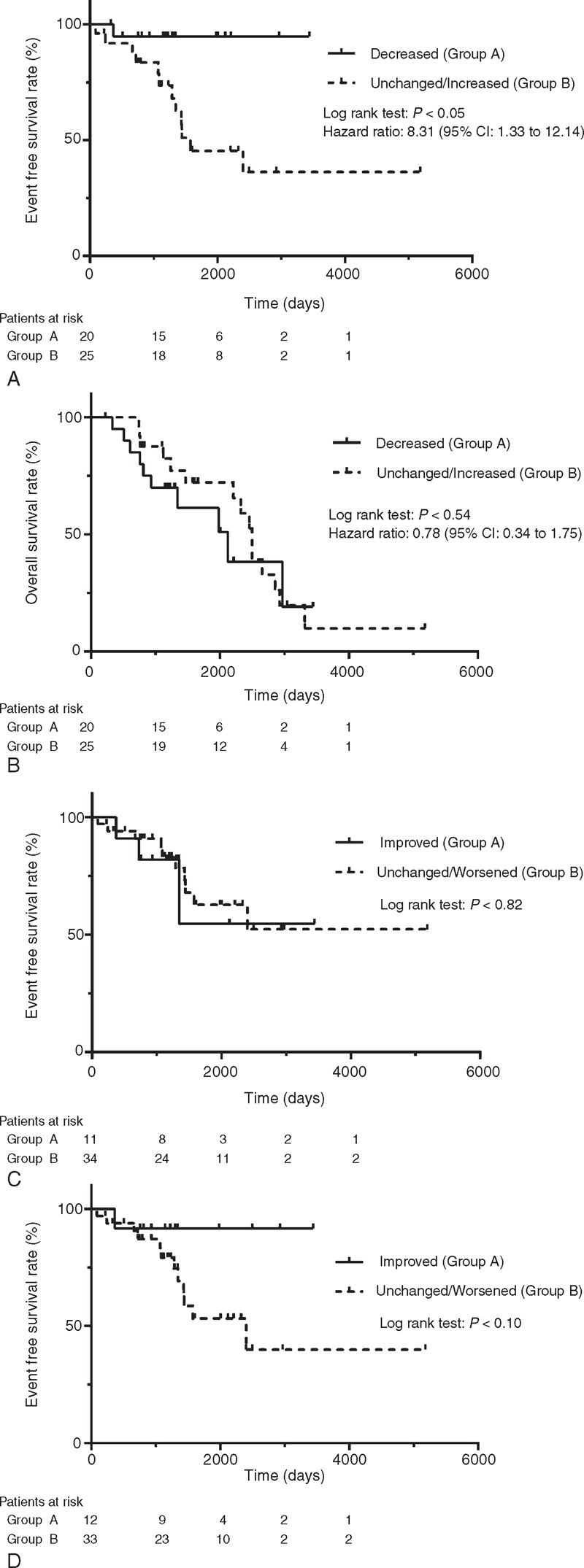
(A) Event free survival for SRE in Group A and B divided by aBSI. Survival curve demonstrated that Group B (Unchanged/Increased) significantly shorter event free survival compared to the Group A (Decreased). (B) Overall survival in Groups A and B. No significant differences were found between the two groups. (C, D) Event-free survival for SRE in Group A and B divided by radiologist interpretation. Survival curves demonstrate no significant differences between Group A (Improved) and Group B (Unchanged/Worsened). Figure 3c illustrates the results obtained by Radiologist A and Figure 3d shows those obtained by Radiologist B.

The median follow-up time was 1342 days. Thirteen patients experienced SRE in the follow-up period, and the median EFS was 1289 days. Kaplan–Meier curves were created for Groups A and B to compare EFS, and a log-rank test showed significantly shortened EFS in Group B (hazard ratio: 8.31, 95% CI: 1.33–12.14, *P* < 0.05) (Figure 3A). No significant differences in EFS were observed between the two groups categorized by the radiologists’ interpretations (*P* = 0.82, *P* = 0.10) (Figure 3C, D).

Twenty patients died during the follow-up period, and the median OS was 1342 days. In OS, the Kaplan–Meier curves showed no significant differences between the 2 groups (hazard ratio: 078, 95% CI: 0.34–1.75, *P* = 0.54) (Figure 3B). Similar to the EFS analysis, no significant differences in OS were obtained between the two groups defined by the radiologists’ interpretations (data not shown).

## DISCUSSION

The present study showed a significant relationship between on-treatment changes in aBSI of bone metastasis and SRE, as well as a correlation between ALP and aBSI. These results suggest that aBSI is useful as an imaging biomarker in bone metastasis treatment for breast cancer.

To date, the relationships between SRE, OS, and BSI have been comprehensively reported in prostate cancer.^10^ On the other hand, corresponding information in breast cancer treatment remains unknown, because few reports exist. Prostate and breast cancer both tend to metastasize to bone, and show similar metastatic sites, that is, the vertebra, ribs, and pelvis adjacent to the trunk. However, an important difference in these two types of malignancy is the bone metastasis pattern, that is, ossification exceeds osteoclastic activity in prostate cancer (osteoblastic bone metastases), while a mixed pattern (osteoblastic and osteolytic bone metastases) is seen in breast cancer. Therefore, the positive results of the aBSI as an imaging biomarker in spite of the different bone metastatic characteristics were interesting as well as promising.

Firstly, the present study demonstrated a significant relationship between the on-treatment changes of aBSI and SRE. Dennis et al^7^ reported similar findings in prostate cancer treatment; the group in which aBSI more than doubled in 3 or 6 months after treatment displayed significantly worse OS, and on-treatment changes in aBSI was considered a response indicator. However, some limitations exist in the present study. Firstly, an irregular follow-up period might have caused a lead-time bias; secondly, flare effects in the bone scan might have altered study results. Performing bone scans under the influence of flare might have caused a temporary worsening of aBSI, resulting in classification of cases as Group B instead of A. Nine patients underwent a scan from 6 to 9 months after treatment started, which appeared to be the period most influenced by flare. Patients who experienced SRE were 1 of 3 in Group A and 3 of 6 in Group B. Six patients in Group B seemed to have been affected by flare by considering aBSI changing in conjunction with serial bone scan findings. Of those 6 patients, only 1 experienced SRE. Considering these facts, flare seemed to have had relatively little effect on the present study results. However, in the future, prospective study with a standardized follow-up period will be needed to avoid a possible flare effect.

Secondly, the present study demonstrated significant differences in EFS but not OS between the two aBSI groups. The median follow-up period of the present study was 44.7 months, which appeared sufficient because generally the reported median OS for breast cancer patients with bone metastasis is approximately 24 months.^11^ However, therapeutic effects of the chemotherapeutic agents or other drugs might have affected the outcome because a long follow-up period spanning years was observed. Therefore, stratifying the subtypes and the treatments is recommended in future analysis of the relationship between aBSI and OS over a long follow-up period.

Thirdly, the present study showed that the radiologists could not predict SRE by using visual scan interpretation, even though the visual scan interpretation results showed a significantly high correlation with the aBSI. The radiologists added 9 or 8 cases into the unchanged/worsened group, compared with the aBSI evaluation. This suggests that radiologists tend to have relatively high sensitivity to the bone scan. This result may stem from an unconscious anxiety about misreading the scan. However, the balance of sensitivity and specificity needs to be adjusted according to the situation, for example, screening test or evaluation after specific treatment. In evaluation after treatment, high sensitivity may lead to false-positive results and physicians may consider more imaging analyses or invasive procedures. Such clinical decisions may considerably burden stage IV patients both physically and economically. Whether the aBSI is superior to radiologist interpretation cannot be concluded from the present study design. Therefore, the aBSI is preferably used as an assistant tool for diagnosing at present. For example, in a facility that has few experienced radiologists, assistant use of the aBSI may enable preservation of the appropriate balance between sensitivity and specificity.

To date, there have been few reliable biomarkers for the evaluation of local therapeutic effect in bone metastatic treatment for breast cancer. Therefore, in clinical settings, tumor markers or bone metabolic markers have been frequently selected because these markers reflect the bone remodeling process. The present study demonstrated a significant correlation between ALP and aBSI. Furthermore, the usefulness of several bone metabolic markers for evaluating the therapeutic effects at bone metastatic sites has been reported, for example, bone specific alkaline phosphatase and procollagen I carboxyterminal propeptide as a bone formation marker, and tartrate-resistant acid phosphatase (TRAPC5b), deoxypyridinoline, and type I collagen cross-linked N-telopeptide (NTX) as a bone resorption marker.^12,13^ However, as often seen in clinical cases, when distant organ and bone metastasis coexist, it may be difficult to separately evaluate a bone metastatic site by serum biomarkers, because the on-treatment changes of each organ to the treatment possibly mimic the changes in the markers. Particularly in such situations, using aBSI as an imaging biomarker will permit objective evaluation of the therapeutic outcome.

aBSI is a promising imaging biomarker, although certain weaknesses, such as the difficulty of eliminating the flare effect, need to be noted. In the near future, combining aBSI with serum biomarkers and utilizing the advantages of each will lead to improved accuracy of therapeutic evaluation.

## CONCLUSIONS

In conclusion, the present study demonstrated the usefulness of aBSI as an imaging biomarker for SRE in bone metastasis treatment of breast cancer. In addition, the combination of the bone metabolic marker with aBSI has the potential to become a powerful evaluation tool. Further investigative analysis is expected to reveal the usefulness of aBSI as an imaging biomarker, similar to its use in prostate cancer.

In the near future, aBSI as an imaging biomarker might be a useful determining factor when deciding between BMA and aggressive orthopedic intervention, thereby leading to decreased SRE.
